# Effects of spinal anaesthesia and intravenous general anaesthesia on the absorption of normal salinein patients undergoing hysteroscopic endometrial resection: an observational study

**DOI:** 10.1186/s12905-023-02404-1

**Published:** 2023-05-09

**Authors:** Wuchang Fu, Xue Li, Hongchun Xu, Ting Zhao, Fangjun Wang

**Affiliations:** 1 From the Second Clinical Medical College of North Sichuan Medical College (Nanchong Central Hospital), Nanchong, 637000 China; 2 From the Affiliated Hospital, North Sichuan Medical College, Nanchong, 637000 China; 3 From North Sichuan Medical College, Nanchong, 637000 China

**Keywords:** Spinal anaesthesia, Intravenous general anaesthesia, Absorption, Uterine distention fluid, Hysteroscopic endometrial resection

## Abstract

**Introduction:**

The absorption of uterine distention fluid during hysteroscopic endometrial resection can cause volumeoverload, which can lead to coagulation dysfunction, acute left heart failure and pulmonary oedema in patients. The effects of spinal anaesthesia and intravenous general anaesthesia on the absorption of normal saline as uterine distention fluid during hysteroscopic surgery remain unclear. The aim of this clinical trial was toobserve the effects of spinal anaesthesia and intravenous general anaesthesia on the absorption of normal saline in patients undergoing hysteroscopic endometrial resection.

**Methods:**

A total of 126 patients undergoing elective hysteroscopic endometrial resection were divided into a spinal anaesthesia group (s group) and a propofol-fentanyl intravenous anaesthesia group (PF group), with 63 cases in each group, and both groups were divided into a short-term group (S_1_ group and PF_1_ group) and a long-term group (S_2_ group and PF_2_ group) according to the operation time. The primary outcome was the absorption of normal saline, and the secondary outcomes included the perioperative SBP, DBP, HR and SpO_2_ and postoperative haematocrit values, and the incidence of postoperative complications.

**Results:**

The volume of saline absorbed was significantly increased in the S_2_ and PF_2_ groups compared with the S_1_ and PF_1_ groups (*P* < 0.001). There was a significant positive correlation between the amount of normal saline absorbed and the operation time (*r* = 0.895,* P* < 0.001). The postoperative haematocrit value was slightly lower than that before the operation in all four groups (*P* < 0.05), and there were no differences in the incidences of urinary retention, sinus bradycardia or hypotension between groups (*P* > 0.05).

**Conclusions:**

There was no difference in the effects of spinal anaesthesia and intravenous general anaesthesia on the absorption of normal saline during hysteroscopic endometrial resection, and the absorption of normal saline increased accordingly with the extension of operation time.

## Introduction

Hysteroscopic surgery has become a standard surgical treatment for abnormal uterine bleeding that is ineffective in conservative treatment, and it has been shown to be a safe and effective alternative to hysterectomy [1]. Hysteroscopic surgery requires distention of the uterine cavity with distention medium to fully display the surgical area. However, distention fluid can be absorbed rapidly through the surgical wound and retained in the body during surgery, which can easily lead to fluid overload. Severe fluid overload can cause coagulation dysfunction, acute left heart failure and pulmonary oedema in patients undergoing surgery [2].

It was found that different anaesthesia methods had different effects on the absorption of glycine as uterine distention fluid during hysteroscopic surgery, but the results were inconsistent [3, 4]. Berg et al. reported that the mean serum sodium level dropped significantly in a monopolar resectoscope using 1.5% glycine with no change in the bipolar resectoscope using 0.9% saline [5], suggesting that bipolar resectoscopes with 0.9% normal saline have a better safety profile. At present, the effects of spinal anaesthesia and intravenous general anaesthesia on the absorption of normal saline during hysteroscopic surgery are unclear. Our objective was to determine whether spinal anaesthesia and intravenous general anaesthesia have different effects on the absorption of normal saline as uterine distention fluid during hysteroscopic endometrial resection.

## Methods

This observational clinical study was performed from February 2022 to August2022, and a total of 126 patients who underwent elective hysteroscopic endometrial resection were included in the study. These women, who had previously been treated with various combinations of progestin, antifibrinolytic drugs, gonadotropin releasing hormone analogues, nonsteroidal anti-inflammatory drugs, and oral contraceptives, underwent endometrial resection due to symptomatic menorrhagia. The inclusion criteria for this study were American Society of Anaesthesiologists (ASA) classification I or II, 18.5 ≤ BMI ≤ 24 kg/m^2^, personal consent of the patient and age 18 to 60 years. The exclusion criteria were coagulation dysfunction, hypertension, diabetes mellitus, deformity in the spinal anatomyor skin infection on the back, history of allergies to local anaesthetics or propofol, submucosal fibroids, uterine prolapse, endometrial hyperplasia, uterine polyps, and cervical or endometrial precancerous lesions. Withdrawal criteria included a change in the surgical plan, refusal by the patient or relatives to continue the study, and incomplete data collection. All patients underwent preoperative transvaginal pelvic ultrasonography, cervical smear and coagulation tests. Patients were grouped according to the anaesthesia methods. If the patients underwent propofol-fentanyl intravenous anaesthesia during surgery, they were included in the propofol-fentanyl intravenous anaesthesia group (PF group). If the patients underwent spinal anaesthesia during surgery, they were included in the spinal anaesthesia group (S group), with 63 cases in each group. According to the operation time, both groups were divided into a short-term group (operation time less than or equal to 30 min) (S_1_ group and PF_1_ group) and a long-term group (operation time more than 30 min) (S_2_ group and PF_2_ group).

The patients fasted for 8 h without any preanaesthetic medication. After arriving in the operating room, all patients were routinely monitored noninvasively for systolic and diastolic blood pressure, electrocardiography, capnography for end-tidal carbon dioxide, pulse oximetry, and heart rate. After good IV access to the upper limb was secured, Ringer’s lactic acidsolution was used for IV hydration during surgery. In both groups, the intravenous fluids were adjusted for fluid maintenance requirements after a bolus of 6 to 8 ml/kg. For patients in the spinal anaesthesia group, a spinal neuraxial block was performed at the L_3-4_ interspace with ropivacaine 15.0 mg (mg) by the anaesthesiologist under an aseptic technique_._ For patients in the propofol-fentanyl intravenous anaesthesia group, general anaesthesia was induced with intravenous administration of midazolam 0.04 mg/kg, fentanyl 2 µg/kg, propofol 2 mg/kg and cisatracurium 0.15 mg/kg. After tracheal intubation, controlled mechanical ventilation was adjusted to maintain an end-tidal carbon dioxide concentration of 40 to 45 mmHg. Anaesthesia was maintained with propofol 4 ~ 6 mg/kg•h and fentanyl 2 ug/kg•h to maintain a BIS value of 40–60 during surgery. All hysteroscopic procedures were performed by an experienced gynaecologic endoscopist with a bipolar resectoscope (Karl Storz SE & Co.KG, Tuttlingen, Germany). Patients were placed in the lithotomy position during the operation, a 0.9% sodium chloride solution was used as the uterine distention medium, and an automatic surgical irrigator (Tonglu Jingrui Medical Instruments Co., Ltd., Zhejiang, China) was used to control the pressure outflow. The uterine distention fluid was irrigated at a variable flow rate under continuous pressure of 100 mmHg. Hypotension (defined assystolic blood pressure falling more than 20% before anaesthesia or systolic blood pressure values lower than 80 mmHg) was immediately treated with an ephedrine 6 mg intravenous bolus. Bradycardia (defined as a heart rate < 55 beats/minute) was treated with 0.5 mg of injected atropine.

The primary outcome was the amount of uterine distention fluid absorbed in each group during the operation. The amount of uterine fluid absorbed was equal to the amount of fluid irrigated into the uterine cavity minus the amount of fluid that passed through the cervix into the container bottle and onto the surgical drapes and the operating room floor. The amount of uterine distention liquid spilled on the floor of the operating room was completely absorbed by the preweighed dry surgical drapes, and then the volume was calculated according to the weight of the liquid absorbed by the surgical drapes and the density of normal saline.

The blood pressure (SBP and DBP), heart rate and pulse oxygen saturation of patients in each group were recorded from a Centricity Anaesthesia system and electronic medical records when the patients entered the operation room (T_0_), 5 min after anaesthesia induction or subarachnoid injection (T_1_), at the beginning of the operation (T_2_), during the operation (T_3_), at the end of the operation (T_4_), and 3 h after the operation (T_5_). A total of 2 ml of arterial blood samples were collected at T_0_, T_4_ and T_5_ to measure the arterial blood gas analysis and haematocrit (HCT) of patients. For each patient, age, body weight, ASA physical status, uterine size, operation time, and intraoperative and postoperative complications such as bradycardia, hypotension, nausea and vomiting, and urinary retention were recorded from electronic medical records using a standardized form.

### Statistical analysis

We calculated that a sample size of 28 patients would be needed in each group (type I error of 0.05, power of 0.9) based on a previous study [6] using PASS 15. Considering a 20% dropout rate, a total of 135 patients were necessary. The following formula was used to compute the sample size:

$${\mathrm{n}}_{ij}=\frac{{\left({\mathrm{Z}}_{1-\alpha /(2T)}+{\mathrm{Z}}_{1-\beta }\right)}^{2}\times \left({{\sigma }_{1}}^{2}+{{\sigma }_{2}}^{2}\right)}{{{\delta }_{ij}}^{2}}$$

$$n=\max\left\{n_{ij},pairs(i,j)\right\}$$

Where n_ij_ represents the sample size of each group, T represents the number of comparisons between the two groups, and σ_1_ and σ_2_ represent the standard deviations of Group 1 and Group 2, respectively. δ_ij_ represents the value of the difference between any two groups with clinical significance. Furthermore, σ was 62 in all groups, μ_1_ and μ_2_ were 100 in the S_1_ and S_2_ groups, and μ_3_ and μ_4_ were 145 in the PF_1_ and PF_2_ groups, respectively.

Data were statistically processed using the SPSS 24.0program. The results were expressed as the mean ± standard deviation (SD) unless otherwise indicated. One-way analysis of variance (ANOVA) with Bonferroni’s post hoc test was used to compare mean differences between groups for demographic data (age, weight, and uterine size), the amount of normal saline irrigated and absorbed, intraoperative intravenous infusion volume and urine volume, and operation time. SBP, DBP, HR, SpO_2_ and HCT were analysed by repeated measures analysis of variance, and the SNK post hoc test was performed if the comparison between groups was positive. *X*^2^ or Fisher’s exact tests were used to compare differences between groups for ASA physical status classification and the incidence of bradycardia, nausea and vomiting, hypotension, and urinary retention. A *P* value of < 0.05 was considered statistically significant.

## Results

**Fig. 1:**
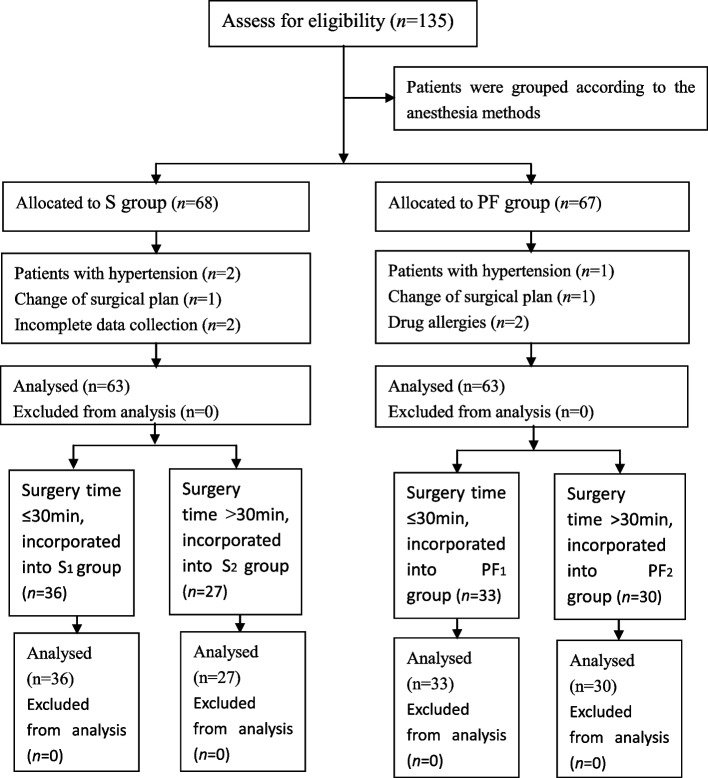
Study flow diagram

**Fig. 2:**
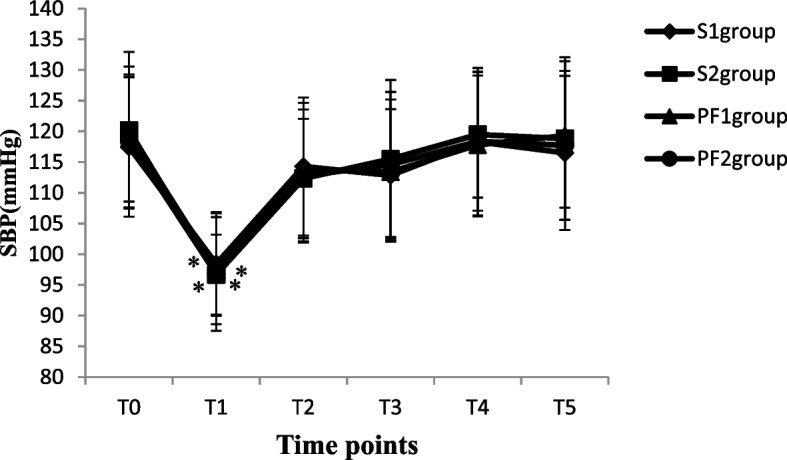
The values of systolic blood pressure in four groups at different time points

**Fig. 3:**
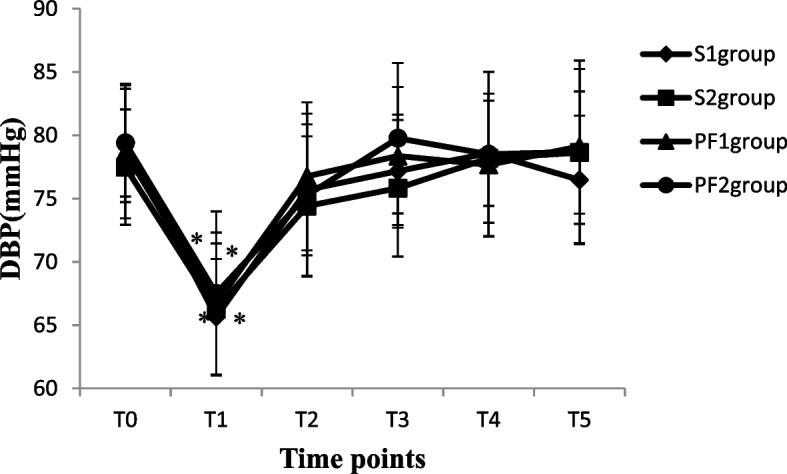
The values of diastolic blood pressure in four groups at different time points

**Fig. 4:**
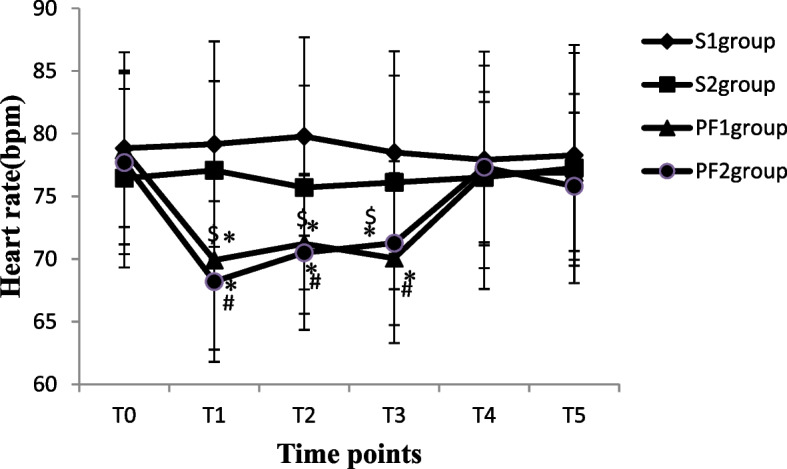
The values of heart rate in four groups at different time points

**Fig. 5:**
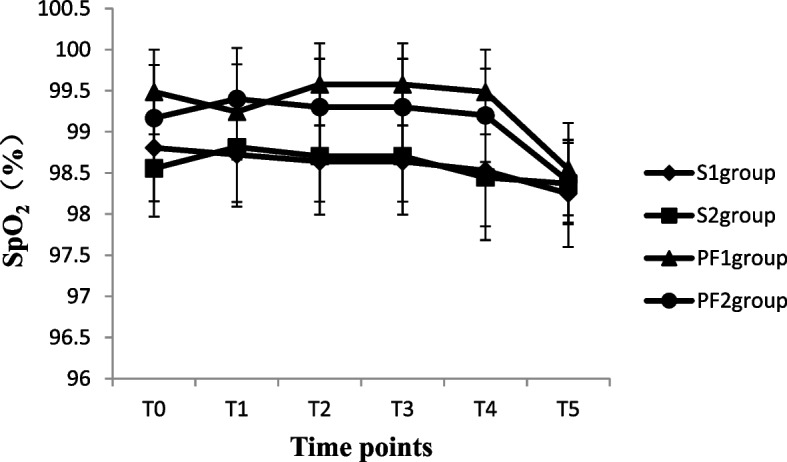
The values of pulse oxygen saturation in four groups at different time points

A total of 135 patients who underwent thysteroscopic endometrial resection were enrolled in this study; three patients with hypertension were excluded (one patient in the PF group and two patients in the S group), two patients in the S group had incomplete data collection, and two patients in the PF group cancelled the surgical procedure due to drug allergies. During the operation, two patients underwent changes in the surgical plan because of intraoperative uterine perforation. Finally, the data of 126 patients were included. There were 36 patients in the S group and 33 patients in the PF group with operation times ≤ 30 min, so 36, 27, 33, and 30 patients in the S_1_, S_2_, PF_1_ and PF_2_ groups, respectively, were analysed (Fig. 1). The demographic data of the patients in all groups were comparable in regard to age, weight, uterine size and ASA physical status classification, as shown in Table 1 (*P* > 0.05). The volume of normal saline irrigated and absorbed, intraoperative intravenous infusion volume, urine volume, and operation time were significantly increased in the S_2_ and PF_2_ groups compared with the S_1_ and PF_1_ groups (*p* < 0.05). The absorption of normal saline was significantly positively correlated with the operation time (*r* = 0.895, *P* < 0.001) (Table 2). After anaesthesia induction or subarachnoid injection, the SBP and DBP values decreased significantly in all four groups (*P* < 0.05). Although oxygen saturation increased significantly from anaesthesia induction to 3 h after the operation in the PF_1_ and PF_2_ groups (*P* < 0.05), it was not clinically significant. The heart rate at T_1~3_ was significantly decreased in the PF_1_ and PF_2_ groups compared with the S_1_and S_2_groups (*P* < 0.05). There were no differences in the values of SBP, DBP or SpO_2_ between groups at different time points (*P* > 0.05) (Figs. 2, 3, 4 and 5). The haematocrit values decreased significantly at the end of the operation (*P* < 0.05) and returned to almost baseline levels3 h after the operation in all four groups. There were no differences in the haematocrit values between groups at different time points (*P* > 0.05) (Table 3). The incidence of postoperative nausea and vomiting was higher in the PF_1_and PF_2_groups than in the S_1_ and S_2_ groups (*P* < 0.05). There was no difference in the incidence of intraoperative or postoperative bradycardia or hypotension (*P* > 0.05). There were two patients with postoperative urinary retention in the S_1_ and S_2_ groups and none in the PF_1_ and PF_2_ groups (Table 4).

**Table 1:** Demographic data

Groups	*n*	Age(y)	Weight(kg)	ASA(I/II)	Uterine size(cm)		
					vertical diameter	anteroposterior diameter	transverse diameter
S_1_	36	37.6 ± 4.3	54.7 ± 3.9	22/14	6.8 ± 0.5	3.7 ± 0.3	4.7 ± 0.4
S_2_	27	35.8 ± 4.6	56.3 ± 4.2	18/9	6.6 ± 0.3	3.6 ± 0.2	5.0 ± 0.5
PF_1_	33	36.6 ± 5.2	54.3 ± 4.3	20/13	6.9 ± 0.5	3.8 ± 0.3	4.9 ± 0.4
PF_2_	30	37.2 ± 4.8	55.1 ± 3.7	19/11	6.8 ± 0.4	3.7 ± 0.3	4.8 ± 0.4
*F*/*x*^2^ *values*		0.549	0.912	0.875	1.308	1.276	0.779
*P values*		0.946	0.437	0.831	0.275	0.286	0.508

Values are mean ± SD, number of patients. S_1_: spinal anesthesia with operation time ≤ 30 min; S_2_: spinal anesthesia with operation time > 30 min; PF_1_: propofol-fentanyl intravenous anesthesia with operation time ≤ 30 min; PF_2_: propofol-fentanyl intravenous anesthesia with operation time > 30 min

*ASA* American Society of Anesthesiologists

**Table 2:** The amount of normal saline irrigated and absorbed, intraoperative intravenous infusion volume, urine volume and operation time

Groups	n	Absorption of normal saline(ml)	Irrigation of normal saline (ml)	Intravenous infusion(ml)	Operation time(min)	Urine volume(ml)
S_1_	36	317.8 ± 16.8	4118.6 ± 287.5	428.7 ± 78.3	25.8 ± 1.6	224.8 ± 43.4
S_2_	27	415.8 ± 14.7^*#^	6215.8 ± 496.6^*#^	514.1 ± 66.4^*#^	53.6 ± 4.4^*#^	316.5 ± 48.8^*#^
PF_1_	33	307.2 ± 11.2	4087.2 ± 356.6	436.2 ± 50.0	26.3 ± 2.0	231.4 ± 50..7
PF_2_	30	421.2 ± 13.7^*#^	6186.2 ± 467.9^*#^	523.6 ± 56.8^*#^	52.5 ± 5.4^*#^	323.7 ± 57.7^*#^
*F values*		338.280	171.743	13.177	413.790	24.313
*P values*		0.000	0.000	0.000	0.000	0.000

Values are mean ± SD. S_1_: spinal anesthesia with operation time ≤ 30 min; S_2_: spinal anesthesia with operation time > 30 min; PF_1_: propofol-fentanyl intravenous anesthesia with operation time ≤ 30 min; PF_2_: propofol-fentanyl intravenous anesthesia with operation time > 30 min

*p < 0.001 vs. S_1_ group, ^#^*p* < 0.05 vs. PF_1_ group

**Table 3:** The values of hematocrit at different time points

Groups	n	T_0_	T_4_	T_5_	*F* values	*P* values
S_1_	36	39.6 ± 2.4	35.8 ± 1.9^*^	38.6 ± 2.3	15.808	0.000
S_2_	27	38.5. ± 2.3	34.9 ± 2.0^*^	37.6 ± 2.4	11.541	0.000
PF_1_	33	39.0 ± 2.6	35.2 ± 1.8^*^	38.1 ± 2.3	15.624	0.000
PF_2_	30	38.8 ± 2.8	36.1 ± 2.4^*^	37.9 ± 2.5	4.626	0.012
*F* values		0.556	1.174	0.429	-	-
*P* values		0.645	0.323	0.733	-	-

Values are mean ± SD. T_0_: before operation; T_4_: the end of operation; T_5_: 3 h after operation. S_1_: spinal anesthesia with operation time ≤ 30 min; S_2_: spinal anesthesia with operation time > 30 min; PF_1_: propofol-fentanyl intravenous anesthesia with operation time ≤ 30 min; PF_2_: propofol-fentanyl intravenous anesthesia with operation time > 30 min

^*^P < 0.05 vs. T_0_

**Table 4:** Intraoperative and postoperative bradycardia and hypotension, and postoperative nausea and vomiting and urinary retention

Groups	n	Nausea and vomiting	Bradycardia	Hypotension	Urinary retention
S_1_	36	3	6	4	2
S_2_	27	3	4	3	2
PF_1_	33	7	5	5	0
PF_2_	30	7	6	4	0
*X*^2^ values		3.896	0.361	0.333	4.303
*P* values		0.043	0.948	0.954	0.231

Values are number of patients. S_1_: spinal anesthesia with operation time ≤ 30min; S_2_: spinal anesthesia with operation time > 30 min; PF_1_: propofol-fentanyl intravenous anesthesia with operation time ≤ 30min; PF_2_: propofol-fentanyl intravenous anesthesia with operation time > 30 min

## Discussion

In our study, there was no difference in the absorption of uterine distention fluid during hysteroscopic endometrial resection using normal saline as the uterine distention fluid between patients undergoing spinal anaesthesia and those with intravenous general anaesthesia. With the extension of operation time, the absorption of uterine distention fluid in patients undergoing spinal anaesthesia or intravenous general anaesthesia increased accordingly. The perioperative haemodynamics in patients during spinal anaesthesia and general anaesthesia were stable. The incidence of postoperativenausea and vomiting was higher in patients with intravenous anaesthesia than in those with spinal anaesthesia.

Because of its low trauma, short operation time and rapid postoperative recovery, hysteroscopic surgery is widely used in the clinic [7]. Different dilatation media are often selected according to the electrodes used in the operation. Fluid overload caused by the absorption of uterineirrigation fluid is the main source of complications during hysteroscopic procedures [8]. At present, there are few studies on the absorption of uterine distention fluid during hysteroscopic surgery. A clinical studyshowed thatthe amount of glycine absorbed with epidural anaesthesia (648.3 ± 157.1 ml) was significantly higher than that with intravenous anaesthesia (380.8 ± 158.2 ml) during endometrial resection. This is mainly due to the expansion of peripheral blood vessels during epidural block, which is more likely to promote the absorption of glycine [3]. Bergeron et al. reported that the absorption of glycine under cervical local block combined with intravenous sedation in endometrial resection (33 ~ 45 ml) was significantly lower than that under intravenous anaesthesia (125 ~ 300 ml). The relaxation of arteriole muscles under general anaesthesia may result in the expansion of systemic arterial blood vessels and accelerate the absorption of glycine [9]. Darwish AM et al. found that there was no difference in the absorbed fluid volumes of glycine and normal saline during hysteroscopic myomectomy under general anesthesia [10]. It was suggested that different anaesthesia had significant effects on the absorption of uterine distention fluid during hysteroscopic surgery, while different dilatation media had no effects on the absorption of uterine dilatation fluid. In our study, we found that there was no difference in the effects of spinal anaesthesia and intravenous general anaesthesia on the absorption of normal saline as uterine distention fluid in either the short-term group or the long-term group, which was inconsistent with the above research conclusions. The reason for this inconsistency may be that the body’s blood vessels were expanded under spinal anaesthesia and general anaesthesia [9], which may have the same effect on the absorption of normal saline during hysteroscopic endometrial resection. In our study, the absorption of normal saline under spinal anaesthesia and intravenous anaesthesia was significantly increased in the long-term group compared with the short-term group. This result indicated that the absorption of distention fluid increasedaccordingly with the extension of operation time [11]. Therefore, the absorption of uterine dilatation fluid should be monitored during long-term hysteroscopic surgery to avoid fluid overload [12].

The clinical study found that the amount of glycine absorbed under spinal anaesthesia combined with oxytocin infusion in hysteroscopic surgery was significantly less than that under intravenous anaesthesia, but the MAP of the two groups was 87.0 ± 10.0 mmHg and 87.8 ± 12.7 mmHg (*P* > 0.05), indicating that there was no difference in blood pressure between the two groups [6]. In the present study, we also found that there was no difference in SBP or DBP between groups at different times, and the incidence of hypotension and bradycardia was similar among all groups. It was suggested that there was almost no difference in the effect of general anaesthesia and spinal anaesthesia on the blood pressure of patients during hysteroscopic surgery, regardless of the different absorption of glycine or the same absorption of normal saline. The main reason for this result is that the difference in the absorption of glycine between the two groups was only approximately 560 ml in previous studies [13]. Most of the hypotonic glycine solution absorbed into the vascular system was quickly transferred into the tissue and cells in the body, and the amount of glycine left in the circulatory system was relatively small, which had little effect on the circulation. In our study, the absorption of normal saline during spinal anaesthesia and intravenous anaesthesia was similar, so the effect of the absorption of uterine dilatation fluid on circulation was also the same in both types of anaesthesia. The absorption of uterine dilatation fluid was primarily studied in the present study, while changes in blood pressure were less frequently observed during hysteroscopic surgery. The operation time was within 60 min in our study, and the effects of the absorption of normal saline on blood pressure in long-term hysteroscopic surgery under different types of anaesthesia were not clear.

A previous study showed that the haematocrit was decreased slightly after hysteroscopic surgery [2]. In this study, the haematocrit of patients after surgery was also slightly lower than that before the operation, but the haematocrit returned to the preoperative level 3 h after the operation. This result indicated that the absorption of uterine dilatation fluid during hysteroscopic surgery had little effect on the haematocrit and that the haematocrit recovered rapidly after surgery. There was no difference in the incidence of postoperative nausea and vomiting between the long-term group and short-term group under spinal anaesthesia or intravenous general anaesthesia. However, the incidence of postoperative nausea and vomiting during spinal anaesthesia was 9.5%, while that during intravenous general anaesthesia was 20.6%, which showed that the time of hysteroscopic surgery had no effect on postoperative nausea and vomiting, while the different types of anaesthesia had a significant effect on postoperative nausea and vomiting [14].

There were several limitations in our study. First, the amount of uterine distention fluid that evaporated during the operation was not included in this study. Because all of the patients included in the study were in the same operating room with the same temperature and humidity, the amount of uterine distention fluid lost during the operation due to evaporation should be consistent for each patient. Second, in our preliminary study, we found that the hysteroscopic surgery time of approximately half of the patients was less than 30 min. Therefore, all patients were divided into a long-term group and a short-term group according to operation times less than or equal to 30 min and more than 30 min in the S group and PF group, respectively. The time of hysteroscopic surgery in this study was within 60 min. The effects of spinal anaesthesia and general anaesthesia on the absorption of uterine distention fluid in long-term hysteroscopic surgery remain unclear and need to be studied in the future. Third, the absorption of no electrolytic solution in transurethral prostatectomy damaged the cascade of blood coagulation, resulting in the inhibition of coagulation factor activity or the reduction of coagulation factor concentration through blood haemodilution. In this study, changes in the coagulation system were not studied, but no patients had coagulation dysfunction during or after the operation. The absorption of normal saline under intravenous anaesthesia and spinal anaesthesia had no significant effect on coagulation function in patients undergoing hysteroscopic endometrial resection. Fourth, the administration of oxytocin during hysteroscopic surgery can significantly reduce the absorption of uterine distention fluid [14, 15]. In our study, oxytocin was not administered during hysteroscopic endometrial resection. Because of the short operation time and low absorption of uterine distention fluid, the patients in this study did not have corresponding complications. Considering that the absorption of uterine dilatation fluid was significantly related to the duration of hysteroscopic surgery, oxytocin should be administered appropriately in clinical practice to reduce the absorption of uterine dilatation fluid and avoid the corresponding complications of fluid overload.

In conclusion**,** there was no difference in the effect of spinal anaesthesia and intravenous general anaesthesia on the absorption of normal saline during hysteroscopic endometrial resection, and the absorption of normal saline increased accordingly with the extension of operation time.

## Data Availability

The datasets used and/or analysed during the current study are available from the corresponding author upon reasonable request. Due to ethical reasons, to protect the integrity of the participants, the study data are not publicly available.
